# Nutritional condition and physiological stress levels of elk in the Black Hills, South Dakota

**DOI:** 10.7717/peerj.7185

**Published:** 2019-06-27

**Authors:** Chadwick P. Lehman, Christopher T. Rota, Joshua J. Millspaugh

**Affiliations:** 1 South Dakota Game, Fish, and Parks, Custer, SD, USA; 2 School of Natural Resources, Wildlife and Fisheries Resource Program, West Virginia University, Morgantown, WV, USA; 3 Wildlife Biology Program, W.A. Franke College of Forestry and Conservation, Department of Ecosystem and Conservation Sciences, University of Montana, Missoula, MT, USA

**Keywords:** Nutritional condition, Physiological stress, elk, *Cervus canadensis nelsoni*, Pregnancy, Lactation, Fecal glucocorticoid metabolite, Black Hills

## Abstract

Percent of body fat and physiological stress are important correlates to wildlife demographics. We studied winter percent of body fat and physiological stress levels for a declining elk (*Cervus canadensis nelsoni*) population in South Dakota, 2011–2013. We obtained percent of winter body fat, pregnancy status, lactation status, and physiological stress data from 58 adult females (2+ years old). We compared physiological stress level data from 2011 with data collected from this same herd when elk densities were much higher (1995–1997). Our objectives were to determine percent of body fat during winter, examine if winter body fat was correlated with pregnancy and lactation status, and quantify and compare physiological stress hormone values from elk in the mid-1990s. Probability of being pregnant increased with higher winter nutritional condition, or percent of body fat; whereas females with a higher probability of previously lactating were lower in winter body fat. Mean fecal glucocorticoid metabolite (FGM) levels in 2011 (mean = 47.78 ng/g, SE = 2.37) were higher during summer compared to data collected in 1995–1997 (mean = 34.21 ng/g, SE = 3.71); however, mean FGM levels during winter did not differ between the two time periods. Although summer levels of FGM have significantly increased since the mid-1990s, we caution against any interpretation of increased FGM levels on elk fitness, as it may not infer biological significance. Mean winter percent of body fat of elk was lower when compared to other populations in the west but this difference does not appear to be limiting vital rates and population growth for this elk herd. We recommend future research focus on summer/autumn data collection to provide a more comprehensive understanding of percent of body fat for elk in our region.

## Introduction

Elements of fitness, such as survival and reproductive success, may be tied to nutritional condition and physiological stress, which can in turn affect population dynamics (Harder & Kirkpatrick, 1994; Saltz, White & Bartmann, 1995; Bender et al., 2008; Breuner, Patterson & Hahn, 2008; Bonier et al., 2009a). Nutritional condition or percent of body fat considers nutritional intake and expenditure, which can influence survival and reproduction (Cook et al., 2004, 2013; Parker, Barboza & Gillingham, 2009). Fat stores are an important component of nutritional condition and are related to animal performance, including pregnancy (Cameron, 1994; Cook et al., 2004; Gustine, Parker & Heard, 2007) and overwinter survival (Hobbs, 1989; Cook et al., 2004; Bender et al., 2008).

Winter nutritional condition, especially when indexed by body fat, reflects adequacy of forage quality and quantity (Parker et al., 1999; Cook, Cook & Mech, 2004) and can affect reproduction and survival (Harder & Kirkpatrick, 1994; Cook, 2002; Cook et al., 2004). Winter condition scores for elk (*Cervus canadensis nelsoni*) at a low level (≤2% fat, equivalent to femur fat ≤40%; Cook et al., 2001a) equates to a higher probability of mortality from winter starvation, whereas winter condition scores of 5% or more body fat during February–March, may indicate good to excellent survival probability through the rest of winter (Cook et al., 2004).

Percent of body fat is tied closely with lactation (Cook et al., 2004, 2013). Lactation can heavily influence body fat scores of elk. It has been hypothesized that the striking differences in body fat between lactating and nonlactating females occurs because digestible energy intake is insufficient during summer and autumn to support both lactation and fat accumulation at the same rate as those of nonlactating cows (Cook et al., 2004, 2013). Lactation during winter might account for observed differences in body fat amounts between lactating and nonlactating females, but most likely milk yields after mid-autumn are too low to appreciably affect energy balance and the observed differences in body fat during winter are carried over from summer and autumn foraging conditions (Robbins et al., 1981; Cook et al., 2013).

Human disturbance can influence nutritional condition and physiological stress in elk. Elk may respond negatively to human disturbance particularly in relation to roads (Millspaugh et al., 2001; Montgomery, Roloff & Millspaugh, 2013), which could displace them from preferred habitats and influence diets (Edge & Marcum, 1991; Morgantini & Hudson, 1985). In addition to these behavioral responses, elk may exhibit a physiological stress response, which is an adaptive response intended to promote survival. Predators may also be a source of stress for elk as predicted by the predation stress hypothesis (Creel, Winnie & Christianson, 2009). Predation from puma was documented as the main source of mortality for elk calves (Lehman et al., 2017) and elk may have been exposed to increased stress from puma. Stress is often measured with fecal glucocorticoid metabolites (FGM; Miller, Hobbs & Sousa, 1991; Creel, Creel & Monfort, 1997; Wasser et al., 1997, 2000; Millspaugh et al., 2001), which is assumed to be a proxy for physiological stress. Although a stress response is adaptive, chronic stress may influence immunity to disease, survival, and reproductive performance (Verme & Doepker, 1988; Wingfield, 1988; Dunlap & Schall, 1995). Higher levels of baseline glucocorticoid secretion have been hypothesized to indicate an individual or population in poor condition, and of reduced relative fitness, as compared to individuals or populations with lower levels of physiological stress (Arlettaz et al., 2007; Thiel et al., 2008). This idea is referred to as the corticoid–fitness hypothesis (Bonier et al., 2009b). Unfortunately, it is difficult for biologists to interpret field measured baseline corticoid levels and how it relates to fitness. Previous findings have been diverse (e.g., negative, positive, non-significant) and should caution biologists who would use corticoid levels as proxies for relative fitness (Bonier et al., 2009a, 2009b; Madliger & Love, 2016).

Elk within the Black Hills, South Dakota may be impacted by physiological stress associated with increasing levels of human disturbance and/or predation. Summer visitation in the form of vehicles on roads, backcountry hiking and biking, and use of all-terrain vehicles (ATVs) and utility-terrain vehicles (UTVs) has increased 2.5 times from 1995 to 2016 in our study area (Lehman, 2015; Lehman et al., 2016; United States Department of Agriculture (USDA), 2017, unpublished data). Surveys indicated a large portion of elk within our study population (≈60%) in Custer State Park (CSP) may have been declining from 2003 to 2011. In CSP elk counts went from a high near 1,200 in 2003 to under 200 in 2011 (Lehman, 2015). Further, predation from puma was documented as a primary source of mortality of elk calves and may be a source of physiological stress for elk (Lehman et al., 2017). Managers were concerned with potential causes for this decline including puma predation and increased human disturbance, which could result in higher stress levels, reduced nutritional condition, and perhaps poor pregnancy rates for elk in CSP and the surrounding area. Our primary objectives were to: (1) determine nutritional condition of elk during winter; (2) examine if winter nutritional condition was correlated with pregnancy and lactation status; and (3) quantify physiological stress hormone metabolites in reproductive aged cow elk and compare levels with previously studied elk from the mid-1990s (a growing population) vs. a declining population (2011–2013).

## Materials and Methods

### Study area

**Figure 1:**
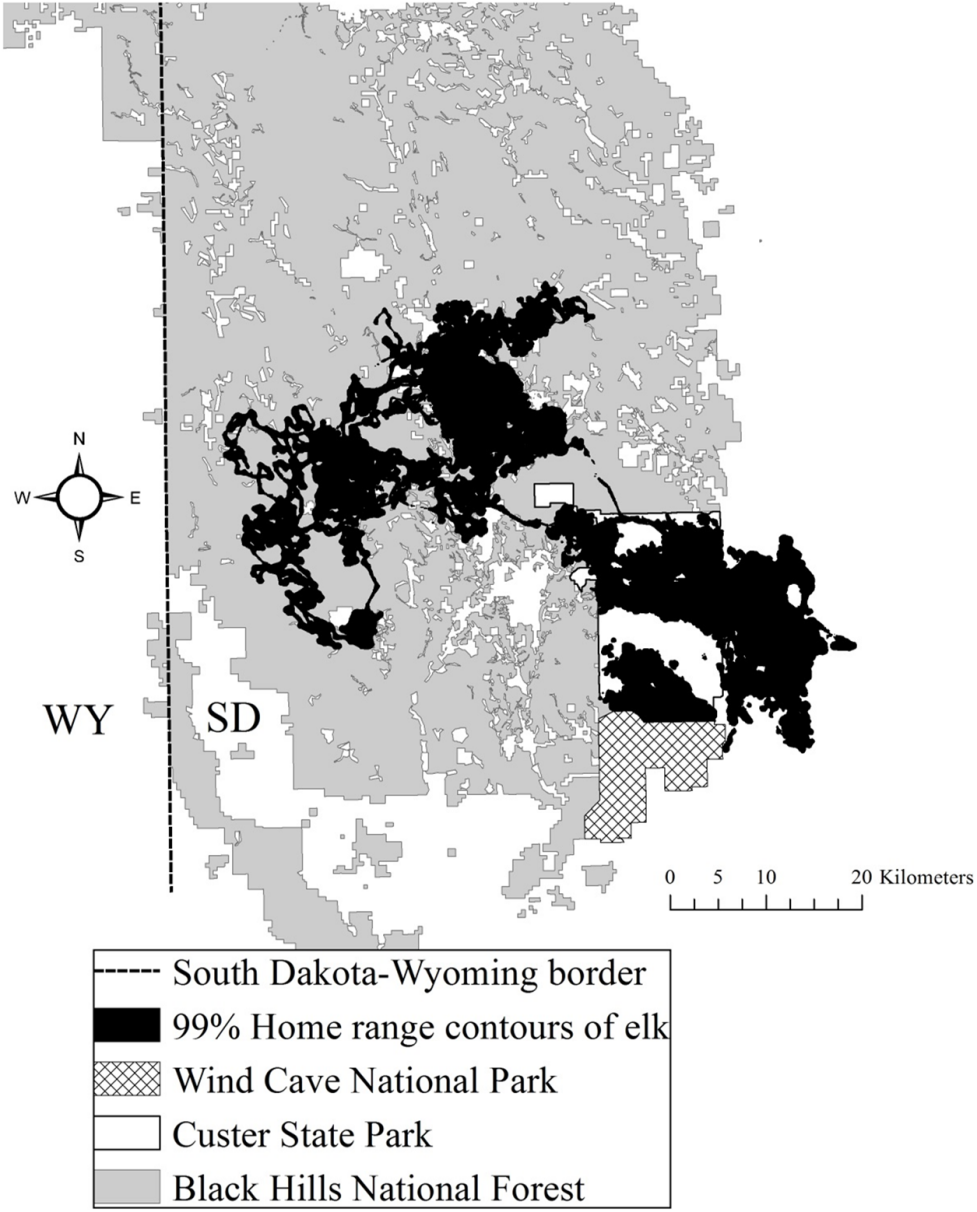
The Black Hills, South Dakota study area where we studied elk nutritional condition and stress, 2011–2013. We provide the spatial distribution of elk using satellite home range locations with 99% Brownian bridge movement model contours.

The study area was situated in Custer and Pennington counties in the southern Black Hills (Flint, 1955). The study area was composed of both public and private land, including CSP, which encompasses 286 km^2^ (Fig. 1). Elevations fluctuated from 1,108 to 2,208 m above mean sea level. From northwest to southeast vegetation varied where forests dominated the central and northern portions of the area at higher elevations and grasslands dominated the southeastern portion of the study area. Mean annual precipitation ranged from 52 to 54 cm and mean annual temperature ranged from 6 to 9 °C across the study area (National Climatic Data Center, 2015). The forests were dominated by ponderosa pine (*Pinus ponderosa*). The eastern portion of the study area was primarily native mixed-grass prairie, agriculture fields, and prairie woodlands (Larson & Johnson, 1999). The average road density in CSP was 2.1 km/km^2^ and the Black Hills National Forest averaged 3.2 km/km^2^ (Lehman et al., 2016).

### Reproductive seasonality

Throughout most of the elk’s range they give birth at the end of May and early June (Hudson & Haigh, 2002). In our study area peak parturition occurs from 28 May to 3 June (Lehman et al., 2016). Elk gestation is 243–250 days with a median of 247 days (Hudson & Haigh, 2002) which would place our peak of breeding at 24–29 September in our study area. For lactation and weaning milk production declines considerably in September and calves are weaned gradually so that nursing bouts are quite infrequent in winter (Hudson & Haigh, 2002).

### Capture and handling

Our study design involved radiomarking and evaluating physiological stress and nutritional condition for an elk herd. This included comparing physiological stress during two time periods, when elk were increasing (1995–1997) and declining (2011–2013). We captured and radiomarked elk as part of two separate studies within the Black Hills, South Dakota, USA, between 1995–1997 and 2011–2013 (Lehman et al., 2016, 2017; Millspaugh et al., 1995). Our techniques used to capture and radio-collar elk from 1995 to 1997 are described by Millspaugh, Brundige & Jenks (1994) and Millspaugh et al. (1995), and from 2011 to 2013 are described by Lehman et al. (2016, 2017). We captured female elk during winter months (January–March) via helicopters. Female elk were randomly chosen from elk herds by selecting animals from the middle vs. near the back of the herd. Once radiomarked, those individuals were captured during subsequent winters to determine winter nutritional condition each year. We monitored most individuals over multiple years leading to greater sample sizes for nutritional condition, pregnancy, and lactation in analyses. We aged females as adults (>20 months) or subadults (≤20 months) using cementum annuli by extracting an upper canine tooth (Matson’s Lab, Milltown, Montana; Hamlin et al., 2000). Our focus was to determine whether percent of winter body fat influences the probability a female will get pregnant and we assessed female elk for pregnancy using rectal palpation during 2011–2013 (Greer & Hawkins, 1967; Vore & Schmidt, 2001). Female pregnancy was determined at 145–155 days of gestation during mid to late February. We drew blood from each collared female to verify pregnancy using pregnancy-specific protein B radioimmunoassays (RIAs; Noyes et al., 1997). We used vaginal implant transmitters to locate and capture elk calves to determine timing of lactation of radiomarked cow elk (Lehman et al., 2017). Our focus was to determine whether percent of winter body fat influences the probability a female will provide milk and wean her calves through mid-September. We assessed lactation of females using their radiomarked calves and survival status as we assumed if calves were still alive they were nursing. Nutritional adequacy of summer range and lactation status the previous summer appear to influence condition of cows much more than lactation during the winter months (Cook et al., 2013). In September milk production declines (Hudson & Haigh, 2002) and if marked calves survived until 15 September the female was classified as having lactated (1), and if not, the female was classified as not lactated (2). Lactation data were collected in 2012 and 2013 when marked calves were monitored in association with their dams. All handling, marking, and monitoring procedures were approved by the South Dakota State University Research Committee (Animal Care and Use Committee Approval Number 11-012A).

### Winter percent of body fat

Nutritional condition or percent of body fat data were only collected on elk from 2011 to 2013. We collected rump nutritional condition scores (rBCS) as described for elk by Cook et al. (2001a). We collected rump fat thickness (MAXFAT) measurements as described by Stephenson et al. (1998, 2002) and Cook et al. (2001a, 2001b, and 2007) via ultrasonography using a University Medical Systems 900 ultrasonagraph with a 5.0-MHz, 7.0-cm probe (Universal Medical Systems, Bedford Hills, NY, USA). We estimated percent of body fat during winter using an arithmetic combination of rBCS and MAXFAT (LIVINDEX; Cook et al., 2001a). Measurements were taken only once per year on elk from mid to late February of each year. Ultrasound training of biologists for this study was conducted by R. and J. Cook at the research facility in LaGrande Oregon, in order to standardize collection methods (Cook et al., 2010).

### Physiological stress

For analysis of physiological stress fecal samples were analyzed using the same lab and methods for both periods (1995–1997 and 2011). For all samples, lab analysis occurred within 12 months of collection. Fecal samples were collected to evaluate FGM hormones from radio-collared elk during winter (16 December–15 February) and summer (1 June–31 August; Millspaugh et al., 2001). We attempted to collect a fresh fecal sample from each individual radiocollared elk at <3-week intervals during both time periods (Millspaugh et al., 2001). We located radiocollared elk by homing on radio signals and visually monitored them until we observed defecation. Late-gestation rise in FGM concentration has previously been reported for elk after 15 March and therefore we did not sample elk during winter after 15 February (Millspaugh et al., 2001; Huber, Palme & Arnold, 2003; Creel, Winnie & Christianson, 2009). Because hormones may be distributed unevenly in feces, we homogenized each sample in the field prior to freezing (Wasser, Risler & Steiner, 1988; Wasser et al., 1996). We froze samples at −20 °C, typically within 2 h of collection (Millspaugh et al., 2001).

We followed laboratory procedures used by Millspaugh et al. (2001) to determine FGM levels. Frozen fecal samples were thawed and we placed ∼10 g subsample in a lyophilizer for 4 days. Once the samples were freeze-dried, we ground them, sifted each through a stainless steel mesh to remove large particles, and thoroughly mixed them. Freeze-drying and grinding preserved FGM, controlled for dietary changes in steroid excretion (Wasser et al., 1994), and allowed for thorough mixing of the sample prior to extraction (Wasser et al., 1996). We extracted glucocorticoids from feces using a modification of Schwarzenberger et al. (1991). We placed dried feces (0.2 g) in a test tube with 2.0 ml of 90% methanol and vortexed at high speed in a multi-tube pulsing vortexer for 30 min. Samples were then centrifuged at 2,200 rpm for 20 min, and the supernatant was saved and stored at −20 °C until assayed (Wasser et al., 2000).

We previously validated a FGM assay for elk using an adrenocorticotropin (ACTH) challenge and parallelism studies in captive elk (Millspaugh, 1999; Wasser et al., 2000). We used 125-I corticosterone RIA kits (ICN #07-120103; ICN Biomedicals, Costa Mesa, CA, USA) to quantify elk FGM concentrations. We conducted parallelism studies to ensure that antibodies accurately measured FGM across their range of concentration. Cross-reactivity of 125-I corticosterone antisera was 100% with corticosterone and <10% for other steroids according to the manufacturer’s report; inter-assay variation for 9 assays was 7.37% and average intra-assay variation was 7.80% (Millspaugh et al., 2001).

We evaluated percent of body fat of elk during winter by comparing LIVINDEX among years (2011–2013) with repeated measures analysis of variance. We used logistic regression to relate percent of body fat with both pregnancy and lactation status as the response variables. We compared FGM (ng/g) concentration levels from 2011 to 2013 with previous levels taken in CSP from 1995 to 1997 using *t*-tests. All analyses were conducted with program R version 3.0.136 (R Core Team, 2017).

## Results

**Figure 2:**
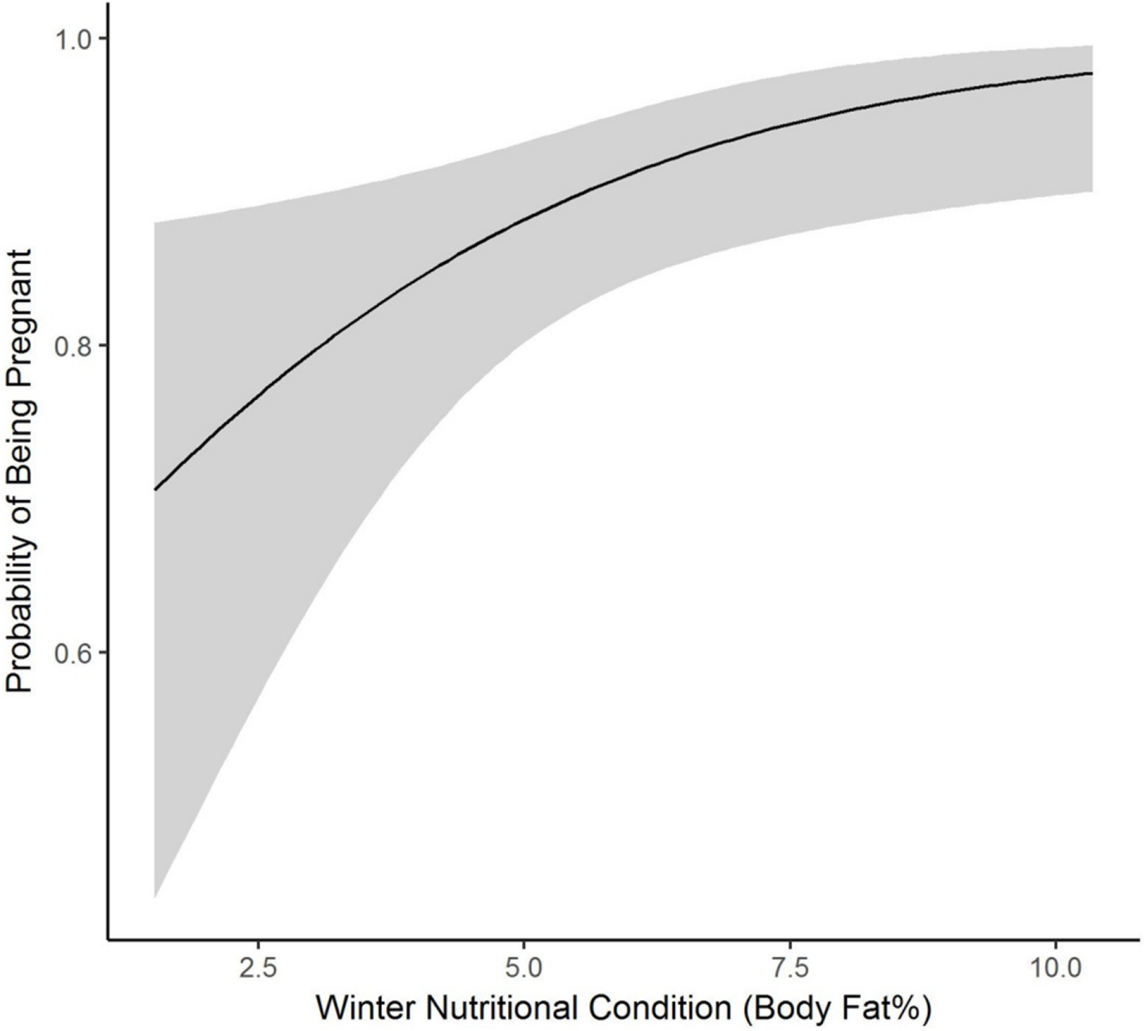
The plotted relationship indicates a slightly increasing probability of being pregnant as winter nutritional condition increases for elk in the Black Hills, 2011–2013, South Dakota.

**Figure 3:**
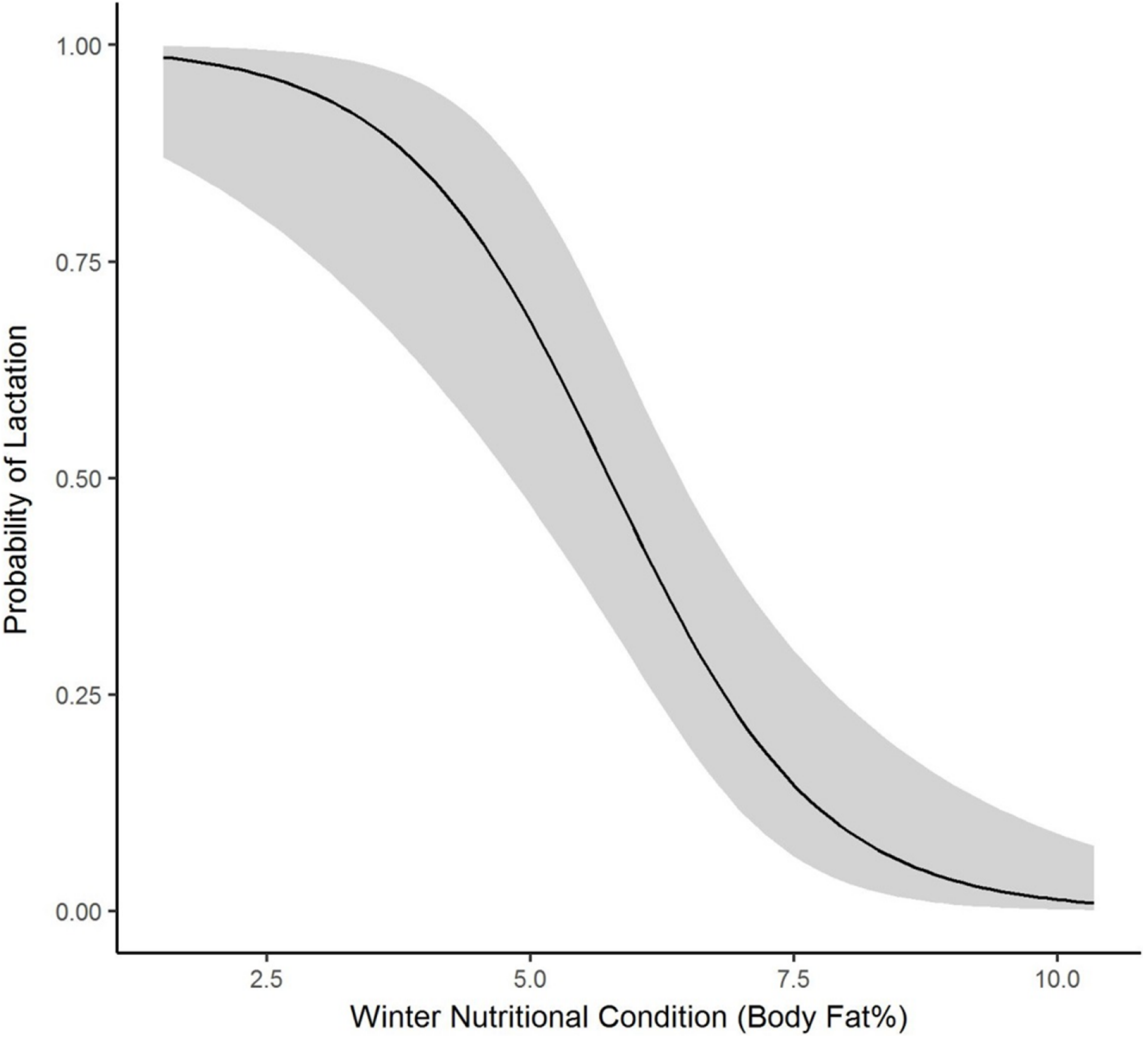
The plotted relationship indicates a decreasing probability of lactating as winter nutritional condition increases for elk in the Black Hills, 2011–2013, South Dakota.

We collected winter percent of body fat data on 58 radiomarked female elk ≥2 years of age between 2011 and 2013. Mean winter percent of body fat was 6.21% (SE = 0.21; Table 1). Winter percent of body fat differed among years (*F*_2,118_ = 6.19, *P* = 0.003), and percent of body fat was highest in 2012 and lowest in 2013 (Table 1). Winter body fat was correlated with pregnancy status (*Z* = 2.39, *P* = 0.02). The plotted relationship indicates a slightly increasing probability of being pregnant as winter body fat increases (Fig. 2). Winter body fat was also correlated with lactation status (*Z* = −4.06, *P* < 0.01). The correlation indicates a decreasing probability of lactating as winter body fat increases (Fig. 3). Females that were not lactating had mean percent of body fat of 7.36 (SE = 0.20) vs. a mean of 4.37 (SE = 0.41) for females that were lactating.

**Table 1:** Mean values and standard errors (SE) for nutritional condition metrics of adult female elk in the Black Hills, South Dakota 2011–2013.

Year	*n*	Body score^a^	SE	Rump fat (mm)^a^	SE	Body fat %^a^	SE
2011	39	2.79	0.13	4.94	0.57	6.10	0.37
2012	40	3.51	0.11	6.04	0.49	7.07	0.20
2013	42	2.66	0.14	4.45	0.52	5.49	0.36
Overall	121	2.98	0.08	5.13	0.31	6.21	0.21

**Note:**

a Nutritional condition (Body fat %), is estimated using the LIVINDEX, an arithmetic combination of body score (rBCS) and rump fat (MAXFAT) (Cook et al., 2010).

**Figure 4:**
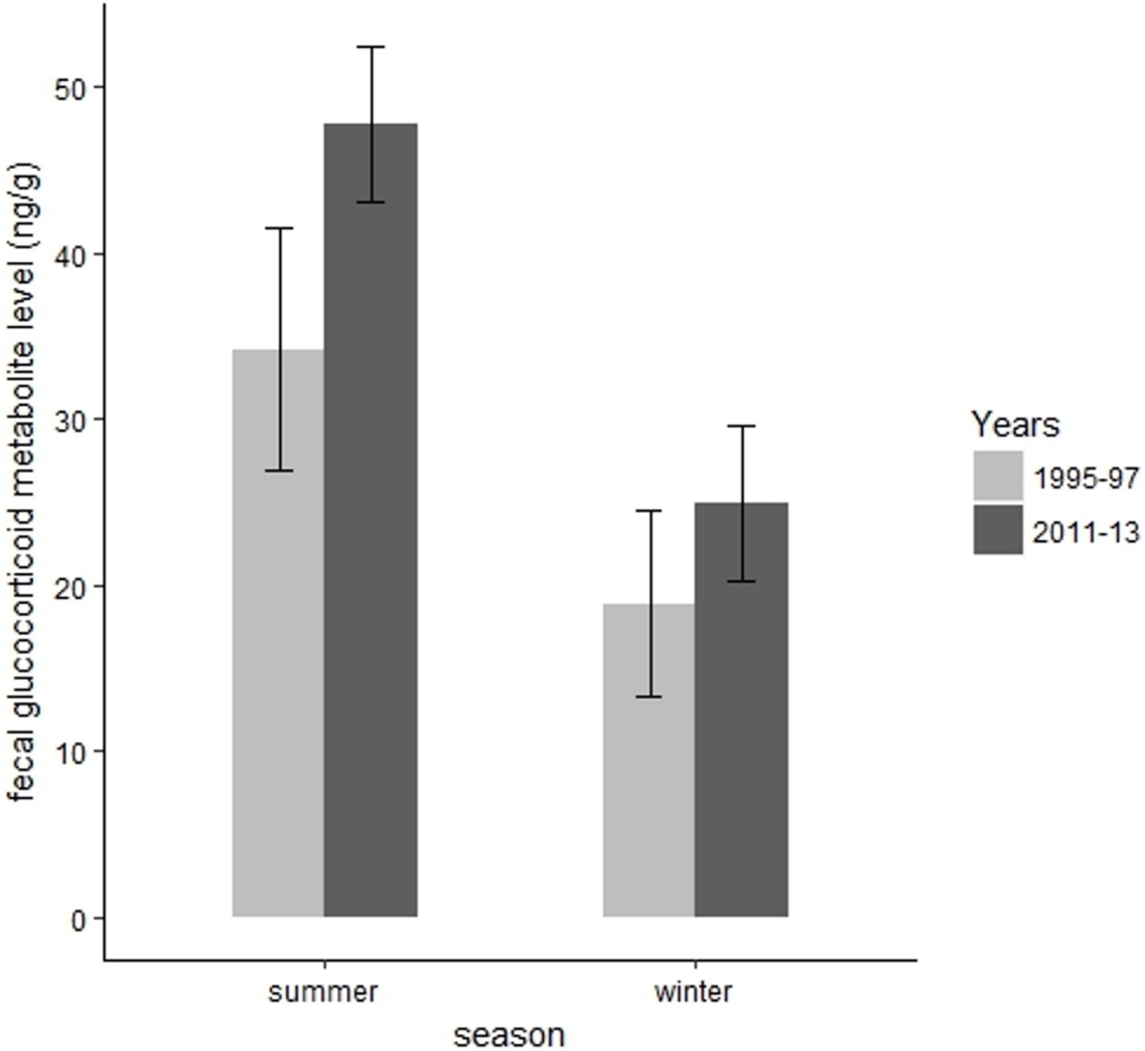
Changes in mean fecal glucocorticoid metabolite levels (ng/g) plus 95% confidence intervals from 1995 to 1997 and 2011 to 2013 for elk in the Black Hills, South Dakota.

We collected 139 fecal samples from 30 radiomarked elk ≥2 years of age during summer months of 1995 and 1997. We only had valid fecal samples from 2011 and we collected 15 fecal samples from 15 radiomarked adult female elk during summer months of 2011. During summer, mean FGM values were 47.78 ng/g (SE = 2.37) during 2011, which was significantly higher than the mean FGM value of 34.21 ng/g (SE = 3.71) during 1995–1997 (*t* = 3.67, *P* = 0.002, Fig. 4). We collected 39 samples from 39 radiomarked elk during winter months of 2011 and 138 samples from 30 radiomarked elk during winter months of 1995–1997. During winter, mean FGM level was 24.94 ng/g (SE = 2.39) during 2011, which was not significantly different from the mean FGM value of 18.90 ng/g (SE = 2.85) during 1995–1997 (*t* = 0.88, *P* = 0.38, Fig. 4).

## Discussion

Winter nutritional condition, or ingesta-free body fat for prime-aged female elk in our study (6.21 ± 0.21) was lower than for other elk populations in the west (Cook et al., 2013). Our data did not include late summer/fall nutritional condition which can be an important indicator of female condition (Cook et al., 2013), but signals were evident among elk and winter body fat percentage in our study. Our design of closely monitoring calf survival allowed us to closely track lactation status of cow elk and lactation status was strongly correlated with winter body fat. Calf mortality from predation was high in this study (Lehman et al., 2017) and females that had calves through fall and winter were much lower in winter body fat compared to females who lost calves earlier in the summer. Similar to our results, nonlactating cow elk had double the body fat of cows that were lactating at the time of capture in Yellowstone National Park (Cook, Cook & Mech, 2004). Lactation puts considerable nutritional demands on elk (Verme & Ullrey, 1984; Oftedal, 1985; Robbins, 1993; Cook, 2002).

Adult pregnancy rates (0.85–0.93) were in the normal range (Raedeke, Millspaugh & Clark, 2002; Raithel, Kauffman & Pletscher, 2007; Lehman et al., 2017) and winter nutritional condition levels were positively correlated with pregnancy rates. Our results followed similar patterns as previous research where probability of pregnancy followed a logistic curve as a function of body fat levels (Gerhardt et al., 1997; Testa & Adams, 1998; Cook, Cook & Mech, 2004). We hypothesize that high calf mortality due to puma predation during the first few months following parturition (Lehman et al., 2017) may allow females to avoid energetic costs associated with lactation, which may in turn improve body fat levels (Cook et al., 2013). A similar pattern was observed in Yellowstone National Park where high calf mortality led to female elk increasing fat levels and pregnancy rates as females did not have the negative energy budget associated with lactation and care of calves (Verme & Ullrey, 1984; Singer et al., 1997; Cook, Cook & Mech, 2004).

Our results parallel previous work conducted in CSP where FGM levels of female elk were higher in summer than in winter (Millspaugh et al., 2001). Winter FGM levels were similar to previous data, but summer FGM levels were higher during our study when compared to data collected from 1995 to 1997 (Millspaugh et al., 2001). Our sampling during winter only encompassed 1 year of sampling in 2011 and our 2012–2013 samples were lost due to spoilage. Although we only provide 15 winter samples from 2011 our standard error was small and the estimate is precise for such a small sample size. Millspaugh et al. (2001) hypothesized that higher FGM levels were associated with high human activity and higher temperatures in summer vs. winter. Mean summer (Jun–Aug) temperatures from 1995 to 1997 (mean = 64°F) were nearly identical with the mean summer temperatures from 2011 to 2013 (mean = 66°F). Therefore, we surmise temperature most likely does not explain the increased FGM levels between time periods. Jachowski et al. (2015) suggested that climatic effects were of lesser importance than human disturbance in explaining elevated stress levels in elk.

However, visitation in CSP during summer has increased since 1995 from roughly 1.2 million visitors in 1995 to 1.6 million summer visitors in 2012 (Lehman, 2015). Disturbance from ATVs and UTVs outside of CSP have increased on lands administered by the USDA Forest Service. Elk in CSP and the Black Hills are exposed to high levels of vehicle and ATV disturbance during spring and summer (Montgomery, Roloff & Millspaugh, 2013). Permit sales for ATVs and UTVs have increased from 7,832 to 19,198 from 2011 to 2016 (USDA, 2017, unpublished data). Snowmobile recreation and increased disturbance was correlated with higher FGM levels for female elk in the Greater Yellowstone Ecosystem (Creel et al., 2002). We hypothesize the increase in visitors and potential activities such as backcountry hiking, biking, and horseback riding, as well as increased ATV and UTV use may have increased summer FGM levels. Further, we hypothesize that the presence of puma and increased predation pressure on calves may have increased summer FGM levels as well. Most of the predation on elk calves occurred from June to August (Lehman et al., 2017) during the same time frame as our FGM sampling. We caution what a summer increase in FGM means relative to fitness and the increases in disturbance may be non-significant for elk demographics. Several studies have collected corresponding vital rate and stress level data, but there is still additional validation necessary to relate these metrics to fitness consequences. Perhaps future manipulative studies that compare how changes within normal baseline FGM levels may change behavior and fitness will further elucidate when FGM–fitness relationships may exist.

## Conclusions

Managers are often challenged to effectively manage habitat to improve and maintain nutritional resources that benefit elk populations, but are often faced with trying to satisfy other natural resource objectives that may not be consistent in meeting objectives of elk habitat quality. To our knowledge, winter body fat levels were among the lowest reported (≈6%) compared with other populations in the west (Cook et al., 2013), but mean growth rates (λ = 1.03), adult survival (90%), and pregnancy (90%) were in the normal range from 2011 to 2015 (Lehman et al., 2017). Further, our stress level data indicate an increasing trend (34.21–47.78 ng/g in summer) but this increase does not appear to be negatively influencing vital rates. In order to determine if there is a nutritional limitation occurring for elk in our study area we recommend that future research focus on summer/autumn data collection, as there is a growing paradigm that nutrition on summer range drives the productivity of elk in many ecological settings (Cook et al., 2013). Future summer/autumn body fat data will provide managers a more comprehensive understanding of elk nutritional condition in our region.

## Supplemental Information

10.7717/peerj.7185/supp-1 Supplemental Information 1 Raw Nutritional Condition Data.

## Additional Information and Declarations

### Data Availability

The following information was supplied regarding data availability: Raw data are available in the Supplemental Files.
